# Annular and Psoriasiform Secondary Syphilis in a Nine-Year-Old Girl Child: A Case Report

**DOI:** 10.7759/cureus.28191

**Published:** 2022-08-19

**Authors:** Aditi A Asia, Deepak Dhadekar, Gauri Sadavarte

**Affiliations:** 1 Medical School, Jawaharlal Nehru Medical College, Datta Meghe Institute of Medical Sciences, Wardha, IND; 2 Dermatology, Shri Vasantrao Naik Government Medical College, Yavatmal, IND

**Keywords:** atypical skin lesions, annular, child, secondary syphilis, sexually transmitted infections

## Abstract

Syphilis, a sexually transmitted infection, may pose a challenge to diagnosis if it presents in an unusual form and in rare areas of the body. Non-typical lesions such as annular, maligna, nodular, nodular-ulcerative, corymbiform, leukoderma, pustular, berry-like, and chancriform presentations comprise about 29.6% of the skin manifestations of secondary syphilis. Although typical secondary syphilis is usually not associated with pruritus, 42% of secondary syphilis patients experience itching. A less frequently seen subtype of secondary syphilis is annular secondary syphilis. Its prevalence is approximately 5.7-13.6%. It occurs more commonly in children and people with dark skin. The location is mainly on the cheeks, frequently near the angle of the mouth. In rare cases, it can occur over the penis, feet, and legs. Syphilis in children is a very rare condition as children are seldom sexually active. Infection can occur through either close contact such as kissing, breastfeeding, vertical transmission, or secondary to abuse. We report a rare case of secondary syphilis having psoriasiform as well as annular lesions manifesting mainly on the palms and soles along with generalized lymphadenopathy in a nine-year-old girl. No evidence of hepatosplenomegaly, icterus, or anemia was seen clinically as well as on sonography. *Treponema pallidum* hemagglutination test was strongly positive. Venereal disease research laboratory test showed a titer of 1:128. Hepatitis B and HIV surface antigen tests were negative. Based on clinical and serological findings, the patient was diagnosed with secondary syphilis, having annular as well as psoriasiform lesions. The patient received tablets of azithromycin 250 mg on the first day. Because of gastritis, the patient was shifted to doxycycline 50 mg twice a day for 14 days. The skin lesions subsided completely after 10 days.

## Introduction

Syphilis is a sexually acquired chronic infection caused by the spirochaete *Treponema pallidum*, named after an afflicted shepherd named Syphilus in 1530. It is distributed worldwide and is seen more commonly in men having sex with men and rarely in children. It is characterized by the involvement of the skin, mucosa, and multiple organ systems [1]. Syphilis goes through several stages if not treated. Typical non-tender indurated ulcer known as chancre develops during primary syphilis. They appear commonly on the genitals at the site of inoculation. Without treatment, the chancre disappears but the disease progresses to secondary syphilis with multiorgan involvement in four to nine weeks [2]. Condyloma lata is a significant clinical finding during secondary syphilis. They are painless, velvety, broad-based, flat, wart-like lesions, which have a tendency to develop in moist, warm sites of the genitals and perineum. The causative organisms, *Treponema pallidum*, which are identified by dark ground microscopy, are abundant in lesions of condyloma lata. If left untreated, it progresses to tertiary syphilis [3]. Transmission of syphilis occurs exclusively during sexual intercourse, including oro-genital contact [4]. However, it can be transmitted by blood transfusion, deep inoculation, vertically from mother to child, or through non-sexual close contact such as kissing and breastfeeding or through accidental puncture with a needle where no primary lesion appears. This type of syphilis is termed “Syphilis d'emblee.” Our case appears to be a case of Syphilis d'emblee with annular as well as psoriasiform lesions in a young girl, which makes it a unique case [5].

## Case presentation

**Figure 1:**
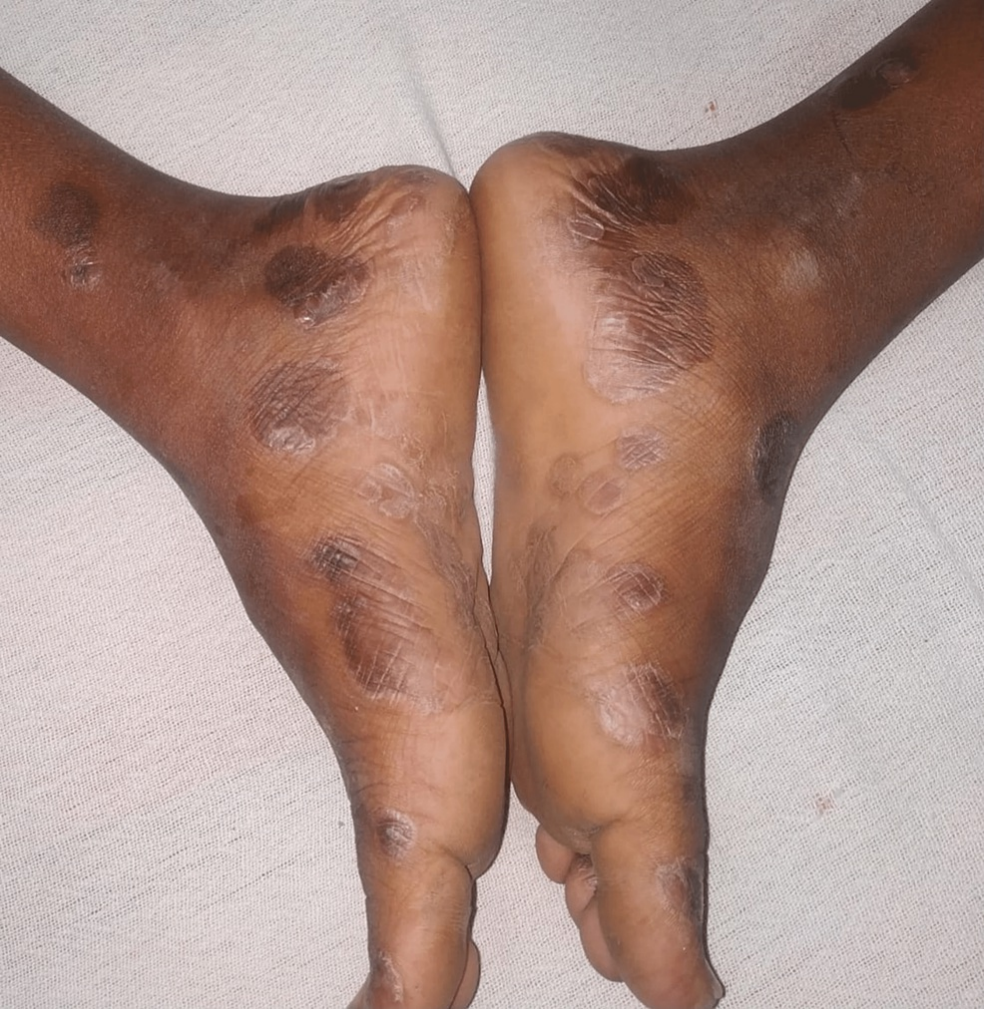
Annular scaly plaques on lower legs and soles.

**Figure 2:**
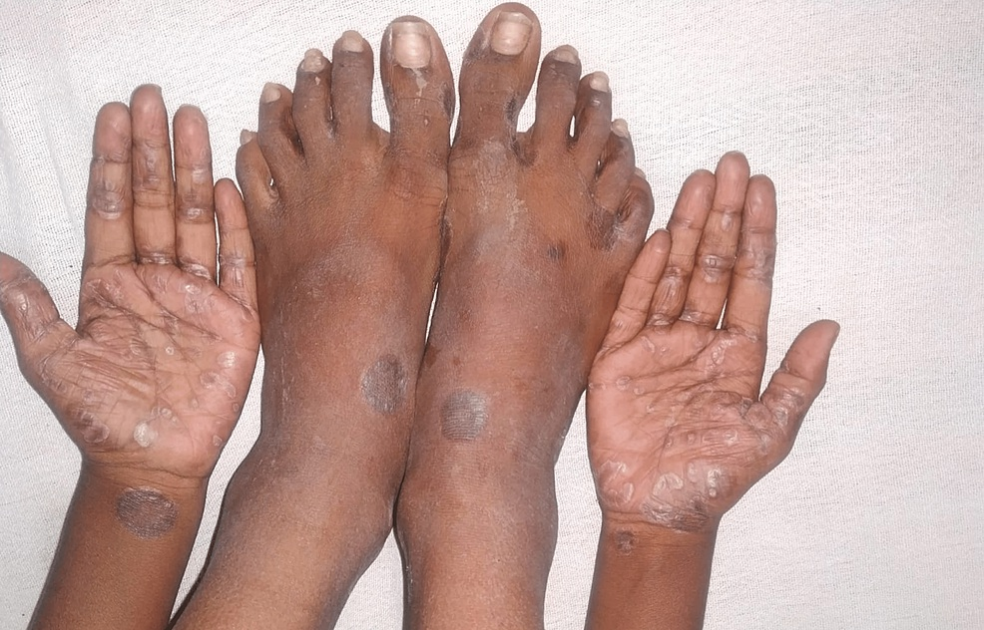
Annular scaly plaques on the palms and dorsum of the foot.

**Figure 3:**
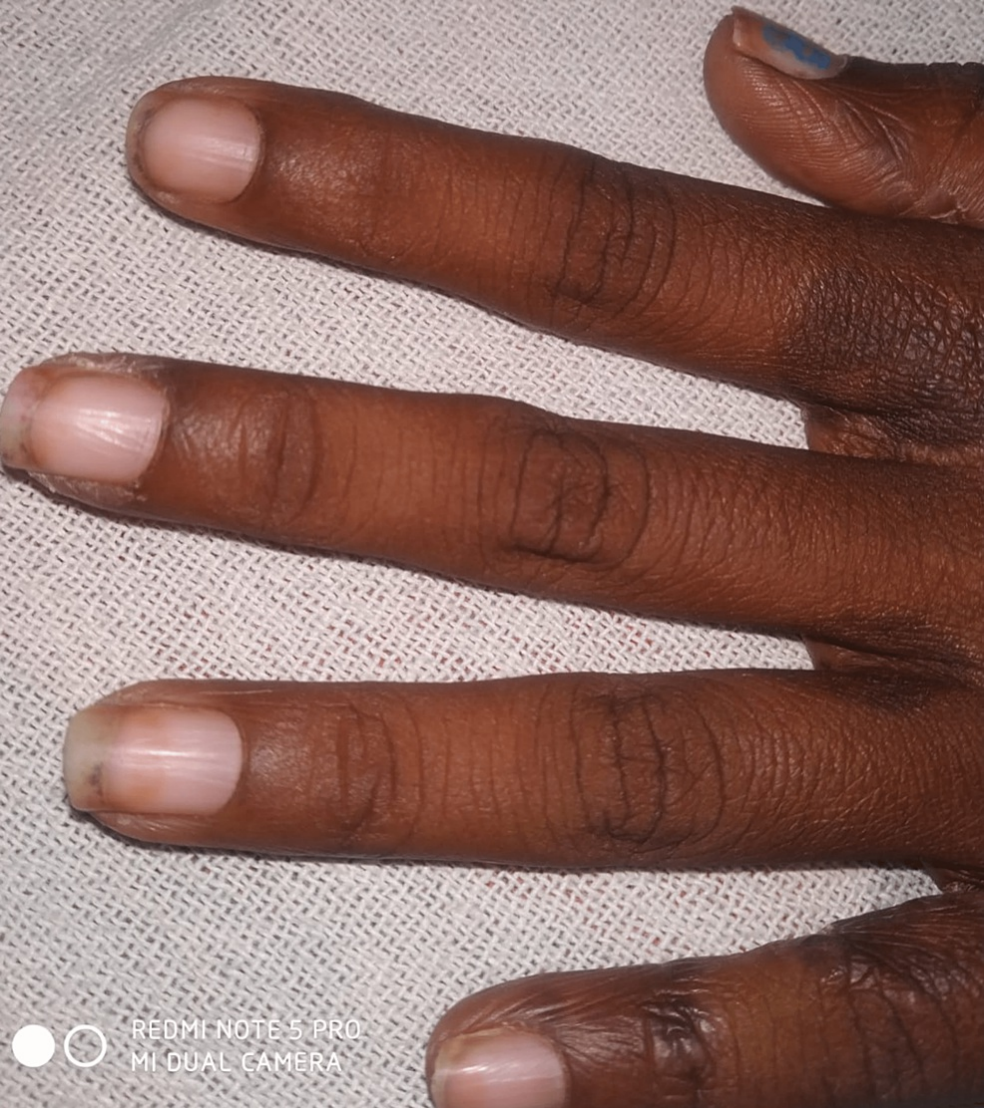
Scales and erosions on lateral nail folds.

A nine-year-old girl from a poor socioeconomic background presented with eruptions on both palms and both soles. The rash was non-pruritic with no history of sore throat, mucosal lesions, or any genital ulcers. The child’s condition was diagnosed as palmar psoriasis by a general practitioner and referred to the Dermatology Department for further evaluation. Skin examination revealed multiple annular, scaly macules and plaques on palms and feet (Figures 1, 2). Nail folds showed small erosions (Figure 3). A single annular plaque was also seen below the nostrils and on the forehead. On examination, bilateral epitrochlear, inguinal, and axillary lymphadenopathy were found. Oral and anogenital areas revealed no similar lesions and were normal without any injuries. The hymen was found to be ruptured. No vaginal discharge was noted. Physical examination showed no evidence of hepatosplenomegaly, icterus, or anemia. Venereal disease research laboratory (VDRL) test showed a titer of 1:128. *Treponema pallidum* hemagglutination test was strongly positive. Hepatitis B and HIV surface antigen tests were negative. Ultrasonography of the abdomen was within normal limits. Chest X-ray was normal. Based on the clinical and serological findings, the patient was diagnosed as having secondary syphilis with annular as well as psoriasiform lesions. The patient received tablets of azithromycin 250 mg on the first day. Because of gastritis, the patient was shifted to doxycycline 50 mg twice a day for 14 days. The skin lesions subsided completely after 10 days.

## Discussion

“”

Overall, 29.6% of patients with secondary syphilis demonstrate non-typical morphology such as pustular, annular, corymbiform, berry-like nodular, nodular-ulcerative, maligna, leukoderma, and chancriform presentations [5-8]. Chapel reported pruritis in around 42% of patients with secondary syphilis [2]. However, it was not reported in our case. A less commonly seen type of secondary syphilis is annular secondary syphilis. Various studies have reported its prevalence to be about 5.7-13.6% [7]. It more often occurs in children and people with dark skin and is primarily seen on the cheeks, often near the angle of the mouth [9,10]. Knöpfel et al reported a rare case of secondary syphilis with a single annular lesion on the scrotum [11]. Our case had annular lesions limited to palms and soles. Reports of psoriasiform lesions are seldom mentioned in the literature; however, Schettini et al. reported a rare case of psoriasiform syphilis having lesions similar to our case [12]. Thorough internal examinations such as X-ray of the chest, sonography, and CT scan did not demonstrate internal organ involvement, as reported by Freitas et al. and others [13]. Differential diagnoses of annular lichen planus, psoriasis, and dermatophyte infection were kept. The absence of pigmentation, oral lesions, and pruritus ruled out lichen planus. The scales in psoriasis are dry, white, and shiny. Because Auspitz’s sign was not observed in our patient, psoriasis was not considered as the likely diagnosis. Non-involvement of intertriginous areas, absence of itching, and no fungal elements on microscopy ruled out dermatophyte infection. Positive serum VDRL and *Treponema pallidum* hemagglutination test suggested recently acquired untreated secondary syphilis in our case. Secondary syphilis shows a good response to penicillin treatment [14]. However, other drugs such as doxycycline, azithromycin, and ceftriaxone are also found to be effective [15,16]. In our case, the skin lesions completely subsided within 10 days with doxycycline. The patient being a minor with a perforated hymen raises the possibility of sexual abuse. The case has been notified to authorities through an STD counselor and a Medical Superintendent.

## Conclusions

With the wide usage of antibiotics, atypical clinical presentations such as annular, psoriasiform, pustular, and nodular lesions are more commonly being noticed. When occurring on the palms and soles, they can be easily misdiagnosed as psoriasis, erythema multiforme, eczema, and annular lichen planus. A high index of suspicion, especially if accompanied by lymphadenopathy, can help in early diagnosis. Although syphilis has been reported to occur through kissing, fondling, and eating prechewed food, sexual contact remains a major route of transmission. Child abuse must be considered in any child presenting with sexually transmitted infection, especially in children with low socioeconomic status and children living in vulnerable circumstances such as children staying in hostels and orphanages. We could not obtain information about sexual activity from the child and parents, yet evidence of a ruptured hymen could be a sign of sexual abuse in our case.
